# Advances in Detection of Antibiotic Pollutants in Aqueous Media Using Molecular Imprinting Technique—A Review

**DOI:** 10.3390/bios12070441

**Published:** 2022-06-23

**Authors:** Akinrinade George Ayankojo, Jekaterina Reut, Vu Bao Chau Nguyen, Roman Boroznjak, Vitali Syritski

**Affiliations:** Department of Materials and Environmental Technology, Tallinn University of Technology, Ehitajate tee 5, 19086 Tallinn, Estonia; akinrinade.ayankojo@taltech.ee (A.G.A.); jekaterina.reut@taltech.ee (J.R.); vunguy@taltech.ee (V.B.C.N.); roman.boroznjak@taltech.ee (R.B.)

**Keywords:** antibiotics, persistent organic pollutants, antibiotic resistance, molecularly imprinted polymers, label-free sensors, environmental monitoring

## Abstract

Antibiotics constitute one of the emerging categories of persistent organic pollutants, characterised by their expansion of resistant pathogens. Antibiotic pollutants create a major public health challenge, with already identifiable detrimental effects on human and animal health. A fundamental aspect of controlling and preventing the spread of pollutants is the continuous screening and monitoring of environmental samples. Molecular imprinting is a state-of-the-art technique for designing robust biomimetic receptors called molecularly imprinted polymers (MIPs), which mimic natural biomolecules in target-selective recognition. When integrated with an appropriate sensor transducer, MIP demonstrates a potential for the needed environmental monitoring, thus justifying the observed rise in interest in this field of research. This review examines scientific interventions within the last decade on the determination of antibiotic water pollutants using MIP receptors interfaced with label-free sensing platforms, with an expanded focus on optical, piezoelectric, and electrochemical systems. Following these, the review evaluates the analytical performance of outstanding MIP-based sensors for environmentally significant antibiotics, while highlighting the importance of computational chemistry in functional monomer selection and the strategies for signal amplification and performance improvement. Lastly, the review points out the future trends in antibiotic MIP research, as it transits from a proof of concept to the much demanded commercially available entity.

## 1. Introduction

Antibiotics constitute a major pharmaceutical agent in medicine. Since the discovery of penicillin, antibiotics have been prescribed to treat and prevent infectious diseases caused by pathogenic microorganisms. In addition, antibiotics are also utilised in agriculture, either as livestock growth promoters or in beekeeping [1,2]. Due to their extensive use, antibiotics are frequently found in aqueous environments at different concentrations, hence their inclusion in the list of environmental pollutants [3,4,5]. This is worsened by the runoff of antibiotic-contaminated farm manure into the surface or groundwater, their ability to escape from wastewater treatment processing and the fact that they could be passed, together with effluents, directly into the river [6,7,8,9,10,11]. Furthermore, development and expansion of antibiotic-resistant pathogens arising from the continuous exposure of microorganisms to sublethal concentrations of antibiotic molecules is a growing concern for many countries [12,13,14,15]. Currently, antibiotic resistance represents a major public health challenge; hence, the World Health Organization (WHO) in 2019 classified it among the ten threats to global health [16]. In addition, it has been estimated that the healthcare systems of European Union (EU) and European Economic Area countries spend about USD PPP 1.5 billion per year in treating over 670,000 antibiotic-resistant bacteria related infections [17,18]. Moreover, apart from the development of virulent strains of microorganisms, the unregulated exposure to antibiotics has demonstrated toxic effects on human, animals and plant lives, resulting in the disruption of vital metabolic processes involving renal and nerve cells, as well as the disruption of plant growth and photosynthesis [19,20,21,22,23]. Although regulatory requirements for the monitoring of antibiotics in environmental water is hard to find, the result of a meta-analysis for the detection of 39 different antibiotics belonging to 9 different antibiotic classes in a variety of aqueous environments and sediments indicated an absolute concentration range between 10^−2^ and 10^6^ ng/L, with the variation directly proportional to the density of human population and agricultural operations. The study also found that the antibiotic environmental concentrations could be above the threshold predicted for driving the development of antibiotic resistant bacteria [24].

To mitigate these challenges, there is a need for routine environmental monitoring of antibiotic pollutants. To these effects, the EU has established a surface water watch list, established in 2015 for deliberate monitoring of chemical pollutants, such as antibiotics, to determine their risk level [25]. Likewise, it is essential to extend the scope of antibiotic surveillance beyond government agencies and equip private individuals with the possibilities of monitoring such pollutants in the comfort of their homes. This will further help to evaluate water before and after treatments and to assess possible contamination all through the water supply chain. However, the achievement of such a goal demands the application of simple, portable, but cost-efficient analytical devices capable of high selectivity and sensitivity, as well as rapid molecular detection. Existing methods for antibiotics detection include chromatography and mass-spectrometry techniques, as well as variations in assays, e.g., immunoassays, microbiological assays, physical and chemical assays, aptasensors and whole-cell biosensors. However, the routine utilisation of these analytical techniques is limited on several fronts, including the demand for a sterile environment, high costs and trained personnel, low portability potential as well as the need for a preliminary sample purification procedure. A critical review of these methods vis-a-vis their adaptability and/or limitations for on-site usage is provided by Parthasarathy et al. [26].

Molecular imprinting is a state-of-the-art technique to generate robust materials with antibody-like abilities to bind and discriminate between molecules [27]. It can be defined as the process of template-induced formation of specific molecular recognition sites in a polymer matrix. In comparison to natural receptors, the main benefits of MIPs are related to their robust nature, i.e., excellent chemical and thermal stability, long shelf life, reproducibility and cost-effective fabrication. Hence, MIPs have shown to be promising alternatives to natural receptors in biosensors. This allows its wide application in nano/biotechnology, chromatography, drug delivery and sensors [28,29,30,31,32,33]. In sensing applications, MIPs are prominently fabricated for numerous numbers of analyte, including macromolecules (Mw > 1000 Da), such as proteins, antibodies, viruses and small molecules (Mw < 500 Da), including most environmental pollutants, mycotoxins and certain biomarkers of pathological conditions [34,35,36,37]. When developing MIP for sensing, the major goal is to ensure the high selectivity of the MIP layer towards the target analyte, allowing the determination of the analyte in the presence of analogous compounds at comparable concentrations [38,39]. In this respect, computer modelling of MIPs has great potential [40]. Moreover, the reliable integration of the MIP layer with a sensor transducer is of great importance. The electropolymerisation [41] and surface-initiated photopolymerisation [42] are apparently the most suitable in-situ synthesis methods for MIP sensing layers. In addition, MIP with a high surface-area-to-volume ratio, which helps to improve the sensor sensitivity, is essential. This is achievable by the development of macroporous MIP layers [43], nanostructured MIPs [44] or MIP composites with nanoparticles (CNT, graphene, etc.) [45].

**Figure 1:**
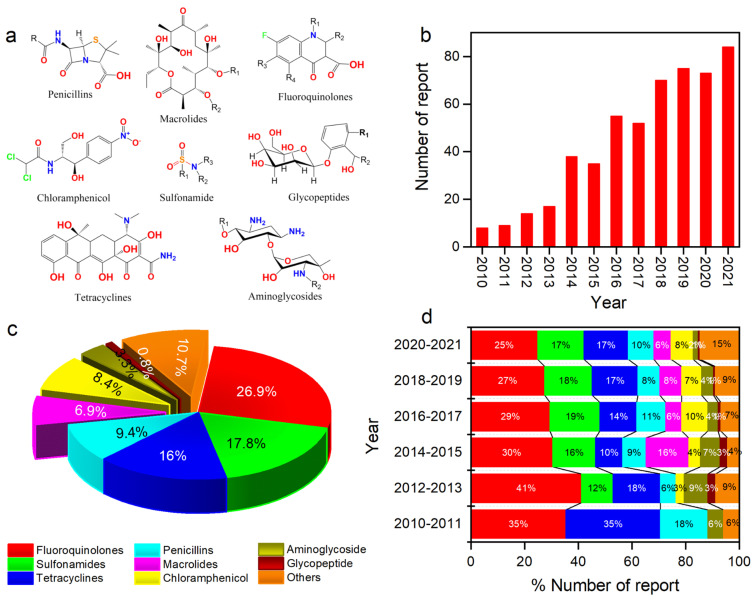
(**a**) Structures of antibiotics belonging to the different classes, (**b**) shares of peer-reviewed articles for different classes of antibiotics between the year 2010 and 2019, (**c**) yearly and (**d**) biannual reports of antibiotic imprinting research between the year 2010 and 2019 (Scopus 4 February 2022).

Antibiotics possess a wide but unique range of chemical functionality around the central atom, which confers their distinct classification (Figure 1a). Fortunately, this functionality can be successfully harnessed to create antibiotic imprints within polymers. When combined with suitable sensing platforms, MIPs are very attractive for routine environmental monitoring of antibiotics considering their low fabrication cost, robust stability and high selectivity. Consequently, within the last decade, reports of MIP-based sensors for antibiotic determination have experienced an upward trend (Figure 1b). In the non-exhaustive list of more than 500 peer-reviewed MIP articles on antibiotic detection in the last decade, it was found that fluoroquinolones constitute the largest share (about 27%) of all antibiotic classes (Figure 1c), exceeding sulfonamides, tetracyclines and penicillins. However, the biannual report (Figure 1d) indicates a declining share of fluoroquinolones, as records of MIP-based sensors for different antibiotic classes continue to gain prominence.

Notwithstanding the increasing research in this field, the commercial implementation of antibiotic MIP-based sensors is yet to be accomplished. The challenges limiting its implementation are discussed in Section 5 of this review, with major preparation issues being incomplete template elution, and robust interfacing of homogeneous MIP layers with sensor transducers. Currently, MIPs have found wide utilisation in the pretreatment of complex samples in chromatographic column, prior to additional MIP-exempted detection. Although the approach is interesting, the multistep processing involved and the undesirable need for an additional detection methodology, which prevents the full exploitation of MIP potential, makes such applications less attractive for sustained adoption. Accordingly, it is critical at this stage to examine the outstanding impacts made within the last decade towards ensuring antibiotic MIP’s commercial implementation and improving their performance. Such a review is essential to answering the following question: ‘how long do we wait to for an antibiotic MIP-based sensor device to be placed on the market?’, while highlighting novel ideas that are fundamental to achieving this urgent aim. Likewise, it is essential to set the record straight to help especially young researchers, who may develop an interest in antibiotic imprinting research, thus fast tracking the development in this field beyond the current state of art. Several review articles currently exist on MIP-based assays for antibiotics [46,47,48,49,50,51,52]; however, the majority of these reviews focus on antibiotic determination in food matrices and/or MIPs intended for use in sample preparation by solid-phase extraction, or as sorbents for separation and preconcentration.

The main task undertaken by this review is to evaluate major trends in the development of MIP-based sensors for detecting antibiotics in environmental water within the last decade, to determine the current state-of-art in the fabrication of point of care testing (POCT) devices for the environmental monitoring of antibiotic pollutants. First, the significance of rational MIP design assisted by theoretical computational modelling for selecting suitable functional monomers for antibiotic imprinting in relation to their analytical performance is investigated. Subsequently, attention is given to the robust integration of MIPs with label-free sensors, including electrochemical, optical, and piezoelectric platforms, as promising alternatives for transforming the scope of antibiotic MIP research from a laboratory-based proof of concept to commercially available analytical tools. Moreover, the review evaluates major approaches for performance improvements, while highlighting probable gaps in this research and recommends new prospects that could intensify efforts towards achieving commercially viable antibiotic MIP sensors in a short time.

## 2. Rational Design of Antibiotic MIPs

The success of molecular imprinting relies on the choice of template, functional and cross-linking monomers, as well as a suitable porogenic solvent. A detailed analysis of the significance of each component was previously reported [53]. In summary, a template could be any molecule, ion, macromolecule, compound or whole cell that has functional moiety that can be harnessed for chemical interactions. Mostly, the target analyte serves as the template during MIP preparation. The polymerisation solvent plays important roles, including solubilisation of all the monomers in the pre-polymerisation mixture, stabilisation of template-monomer complexes and acting as a porogen that helps to control the porosity of the resulting polymer. Following polymerisation, functional groups of the monomers are held in position by cross-linking the polymeric structure, thereby cementing their orientation within the polymer after template extraction.

Indeed, MIP recognition of a target is greatly influenced by the strength of the interaction between the template and functional monomer during the pre-polymerisation complex formation stage that then dictates the binding affinity, usually represented by the dissociation constant (i.e., KD) of the MIP for the targets at rebinding. A lower KD would generally suggest that the recognition layer possesses a greater fraction of high-affinity binding sites, due to a strong interaction (high binding energy) between the template and the monomer that was formed during the pre-polymerisation stage. Moreover, a strong interaction between the template and functional monomer is critical, especially in the analysis of small targets, such as antibiotics, where a high binding affinity (low KD) is required to analyse low concentrations of analytes.

Depending on the interaction type in the template–monomer complex, molecular imprinting approaches are generally categorised as covalent and noncovalent. The covalent approach employs reversible covalent bonds between the functional monomer and template, such as boronate ester, ketal/acetal, and Schiff’s base formation. This strategy leads to the generation of a higher yield of specific and more homogeneous binding sites, while the applicability is limited because of the small number of compounds bearing required functionalities (alcohols (diols), aldehydes, ketones, amines, and carboxylic acids). Another disadvantage of covalent imprinting is the complicated template removal and slow binding kinetics of the resulting MIP. On the contrary, the most frequently employed noncovalent approach offers a wide variety of functional monomers, with high flexibility and rapid rebinding kinetics. This approach is based on noncovalent interactions, such as hydrogen bonding, electrostatic interactions and van der Waals forces in the template–functional monomer complex. In addition, molecular imprinting based on metal-ion coordination was introduced [54,55]. In this approach, the functional monomer and template are bridged through coordination binding with various metal ions, such as Cu^2+^, Ni^2+^, Cd^2+^ or Zn^2+^. Compared to noncovalent interactions, metal-ion coordination bonds are stronger, leading to better stability of the MIP in aqueous media. Hence, the selection of an appropriate functional monomer capable of forming a stable complex with a target analyte via the reversible covalent, noncovalent or metal-ion coordination is of great importance for a successful imprinting process. Notwithstanding, not all monomers fulfilling this obligation are suitable candidates. This is because the appropriate monomer must also be compatible with the desired polymerization approach. Therefore, a potential functional monomer should possess chemical groups for polymerisation and formation of strong interactions with template molecules. Although such a demand may suggest a limitation in the number of suitable functional monomers for molecular imprinting, new functional monomers tailor-made to accommodate these challenges are being synthesised [56,57,58,59,60].

Moreover, cross-linking monomers are essential for controlling the morphology of the MIP matrix during polymerisation by fixing functional monomers around template molecules. Typically, an insufficient amount of crosslinker reduces the structural stability of the polymer, whereas an excess reduces the number of MIP binding sites. Reportedly, most commercial crosslinkers, such as ethylene glycol dimethacrylate (EGDMA), are compatible with molecular imprinting [61]. However, the selection of crosslinkers should be based on their solubility in the synthesis medium and the strength of the interaction with the template. It was reported that the crosslinker that displays lower binding of the template should be preferential because it generates MIPs with lower non-specific binding and a higher imprinting factor, and therefore specificity [62]. If the level of non-specific binding background on the MIP is high, a similar polymeric material, i.e., non-imprinted polymer (NIP) generated in the absence of the template, might serve as a good reference, allowing one to compensate the background and accurately analyse label-free responses originating from a MIP modified sensor.

**Figure 2:**
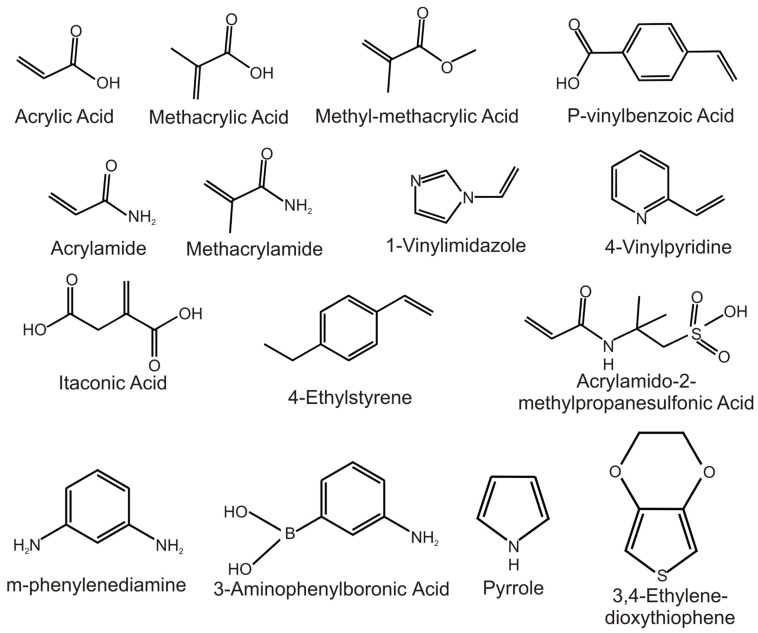
Structures of commonly used functional monomers for molecular imprinting.

Various functional monomers exist for building polymer matrices for molecular imprinting. Figure 2 shows the structure of monomers commonly employed in antibiotic imprinting. Among these, methacrylic acid (MAA) is the most utilised. This is partly because hydrogen bonding constitutes one of the dominant interactions employed in MIP research and MAA can serve as a hydrogen bond acceptor or donor. Moreover, it was revealed that the dimerisation of MAA can improve, to an extent, the success of molecular imprinting [63]. However, the growth in scientific efforts has resulted in an increasing utilisation of other monomers, thereby removing the monopoly in MAA usage.

The understanding of the noncovalent interactions between a template and functional monomer could be obtained through computational software that assists in studying these interactions at the molecular level, thereby allowing the optimisation of factors that affect the performance of the MIP-based system. The adaptation of computational approaches for rational design of MIPs requires calculating binding energies between a template and functional monomer(s) and designing the more specific and selective recognition sites in the polymers [64]. The computational approach demonstrates superior advantages over experimental trial and error due to its time efficiency, low costs, and the possibility to circumvent the use of expensive and toxic chemicals.

In molecular imprinting research, commonly employed computational approaches for estimating noncovalent interactions between a template molecule and a monomer in pre-polymerisation solution include quantum chemical calculations (QCC), molecular docking (MD), molecular mechanics (MM), and molecular dynamics. For small molecular weight analytes, QCC has become the most promising approach for calculating hydrogen binding energies between the template and monomer [65]. To perform a QCC calculation, the geometry of the monomer-template complexes and their individual compounds are optimised by semi-empirical (SE) parameterised method 3 (PM3). This is then followed by the estimation of the binding energy by either density functional theory (DFT) or Hartree–Fock methods. Based on the DFT method that is commonly used to estimate hydrogen binding energy between small molecular-weight templates (e.g antibiotics) and monomers, a hydrogen bond interaction is usually classified as strong (>63 kJ mol^−1^), moderate (16.8–63 kJ mol^−1^), and weak (<16.8 kJ mol−1) [66]. A brief account of antibiotic MIP-based sensing research preceded by a painstaking but rewarding computationally assisted selection of the functional monomer is provided here, while a more comprehensive list is shown in Table 1.

Tadi et al. [67] achieved a rational selection of pyrrole, as the functional monomer for the imprinting of sulfanilamide among many monomers, with the help of QCC and DFT methods. The high binding energy obtained between the template and monomer was traceable to the hydrogen bond formed between S=O and -NH2 groups of the template and the -N and -CH groups of pyrrole. After electrodeposition on a pencil graphite electrode and parameter optimisation, the MIP demonstrated a discriminatory recognition for the target and the assay indicated a significant LOD of 20 nM. Similarly, to select an appropriate monomer for the preparation of amoxicillin MIP on QCM, QCC was employed to estimate the binding energies between the target and several electropolymerisable monomers including pyrrole, meta-phenylenediamine (mPD) and 3,4-ethylenedioxythiophene (EDOT) [68]. The study indicates the highest binding energy for mPD (273 kJ/mol), as compared to 63 and 8 kJ/mol for pyrrole and EDOT, respectively. The significance of this study was reflected in the sensor’s superior binding of the target (about 7 times higher adsorption capacity) over its reference NIP-based sensor.

Although MD and molecular dynamics are conventional in the rational monomer selection for imprinting of macromolecules, e.g., protein, their implementation is extended to small molecular weight templates [69,70]. Thus, in two separate reports [71,72], MD was applied for the rational selection of a monomer for the imprinting of norfloxacin using CDOCKER and SYBYL software. In both cases, MAA gave the highest binding energy among the tested monomers, including acrylamide, methacrylamide, polyvinylpyrrolidone, and MAA. Although the binding energies in both reports were slightly different (100.33 vs. 87.45 kJ/mol), a pronounced difference in the affinity (0.004 vs. 2.06 μM) and relative adsorption capacity (4.3 vs. 2.4) was observed. This further indicates the importance of accounting for molecular interaction between a template and a functional monomer and that a small change in the effectiveness of the interaction might lead to a significant effect on the performance of the MIP-based sensor.

**Table 1:** Antibiotic MIPs designed by computationally assisted selection of functional monomer and their analytical performances in different aqueous media.

Computational Approach	Template	Monomers Studied	Binding Energy (kJ/mol)	Selected Monomer	K_D_ (μM)	Q_MAX(MIP)_/Q_MAX(NIP)_	LOD (nM)	Media	Ref.
QCC	Sulfanilamide	Pyrrole	18.86	Pyrrole	-		20	Ground water	[67]
		Furan	15.11						
		Thiophene	8.62						
		1-Methylthiophene	7.63						
		Methylpyrrole	7.13						
	Flumequine	Pyrrole	34.98	Pyrrole	20	-	1000	Aquaculture water	[73]
	Sulfadiazine and sulfamerazine	AA	557.727 552.58	AA	0.19	>3.0		water	[74]
	Sulfamethizole	mPD Pyrrole EDOT	181.20 80.26 30.10	mPD	47.2	8.24	2	Tap water	[75]
	Amoxicillin	mPD EDOT Pyrrole	273.05 8.40 63.01	mPD	28.8	7.2	0.2	PBS	[68]
	Azithromycin	4-ABA Phenol Pyrrole Aniline Thiophene	71.55 69.41 68.93 45.08 31.96	4-ABA	-	-	80	River water	[76]
Molecular Docking (CDOCKER)	Norfloxacin	AM AA MAM MAA N-iAA PVP	82.22 54.27 58.95 100.33 61.04 76.32	MAA	0.004	4.3	31	Waste water	[72]
	Norfloxacin	MAA AA MAM AM 4-VP	87.45 64.68 53.97 76.65 56.48	MAA	2.06	2.44	16	Lake water	[71]
Molecular Docking (SYBYL)	Cefquinome sulphate	Pyrrole-2-carboxylic acid Pyrrolidine-2-carbohydrazide 4-ABA oPD 4-ATP	603.09 485.79 435.47 325.77 271.94	4-ABA	-	3.5	-	PBS	[77]
	Nafcillin	oPD Proline Aniline Pyrrolidine-2-carbohydrazide 4-Aminobenzoic acid	163.55 159.41 152.67 149.33 135.23	oPD	-	-	80	River water	[78]
	Chloramphenicol	AA, MMA	75	AA	-	-	1.5	Tap water	[79]
Molecular Dynamic	Norfloxacin	MAA/EGDMA ratio is optimised	-	MAA, EGDMA	374; 279 772; 481	High adsorption capacity (29.35 mg/g)	-	Water	[80]
	Sulfamethoxazole	APTES/TEOS ratio optimised	-	APTES, TEOS	-	-	60	Lake water	[81]
Molecular Mechanic	Penicillin G	CMA and CSEV compatibility	-	MAA, TRIM	-	6.03–6.69	-	Water	[82]

## 3. Label-Free Detection of Antibiotics

Analytical chemists are continually faced with the challenge of developing accurate, cost-effective and rapid detection methods for the analysis of substances for different aims, including environmental monitoring. In labelled detection methodologies, such as radioactive, fluorescence and luminescence, specific reagents and/or molecules tagged to a target analyte or a competing molecule are monitored as an indirect indication of analyte detection. In such arrangements, the labels provide the analytical signals. As a critical requirement, a label or the labelling protocol must neither affect target analyte functionality nor compete with analyte binding sites and should not introduce bias between different experimental runs. Moreover, labelling can be time-consuming, expensive and may introduce artefacts that would affect analyte–receptor interactions, thereby compromising analytical integrity. It should also be taken into account that the distribution of labels around the target or competitor may be nonhomogeneous, and the analytical conditions may disrupt label functionality (e.g., fluorescence quenching).

To overcome these challenges, label-free detection provides an alternative approach in which no labelling is required, thus eliminating complexity in analyte detection. This is realised by monitoring the intrinsic phenomenon, including mass, electrical impedance, refractive indices, ionic charges, etc., induced by immediate binding of an analyte to a receptor-modified sensor surface. Thus, with the label-free method, a real-time, rapid, and cost-effective analysis of target analytes can be easily achieved when combined with selective recognition elements. Moreover, label-free detection methods enable a more detailed analysis of binding interactions, including affinity and kinetics, in addition to their multiplexing capacities and the possibilities of fabricating miniaturised lab-on-chip sensing devices. A label-free assay for small molecules, such as antibiotics, initially experienced a setback, especially for technologies that depend on a change in mass to monitor analyte determination. This is due to the small or sometimes non-analytically detectable signals (low signal-to-noise ratio) induced by these low molecular weight analytes. However, recent collaborative efforts from the point of view of both instrumental technological advancement and laboratory research have improved their analysis. Namely, novel methods, several immobilisation strategies and state-of-the-art recognition elements are now being employed.

**Figure 3:**
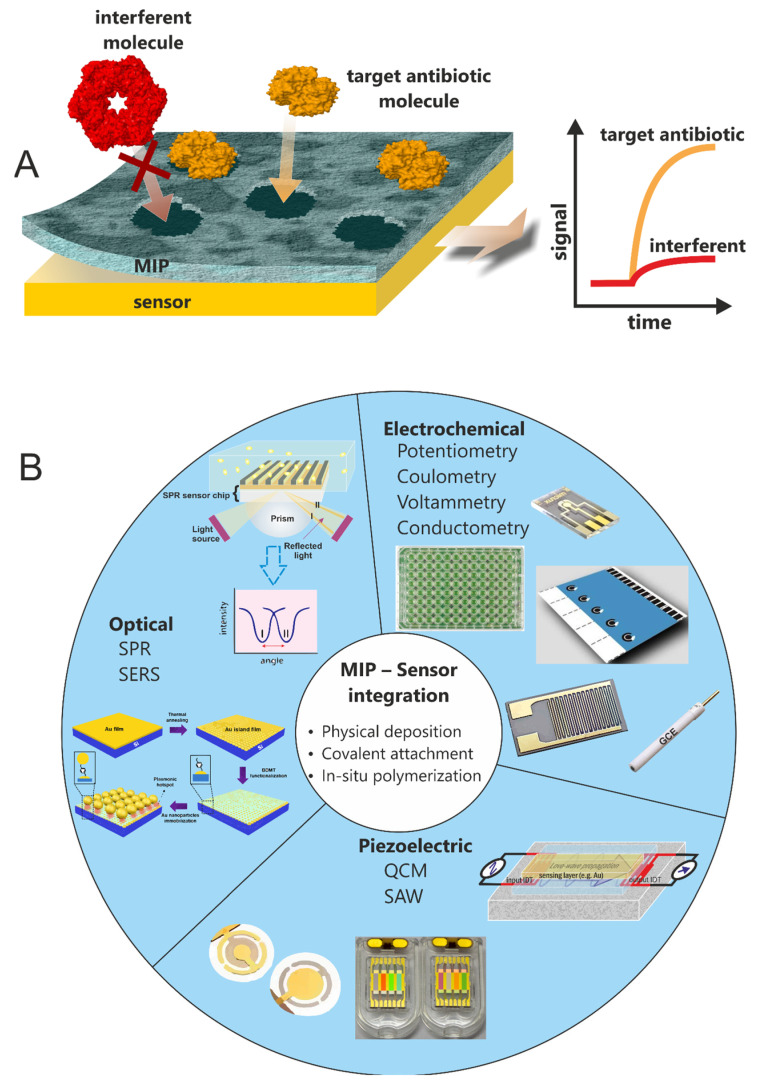
(**A**) Schematic illustration of the label-free detection by a MIP layer integrated with the sensor surface, (**B**) types and examples of label-free sensors commonly employed in MIP research. SERS example is reproduced with permission from Springer Nature (doi.org/10.1038/s41598-021-01742-0).

In molecular imprinting research, MIP layers are commonly integrated with the sensing surface of a transducer belonging to different label-free sensing platforms (Figure 3). Such platforms include optical (e.g., surface plasmon resonance (SPR), surface-enhanced Raman spectroscopy (SERS), etc.), piezoelectric or mass sensitive (e.g., quartz crystal microbalance (QCM), surface acoustic wave (SAW)) or electrochemical (Au/C electrodes, pencil graphite electrode, screen printed electrodes (SPEs), carbon nanotubes (CNTs), etc.) platforms. The integration of MIP layers with label-free transducer surfaces may be achieved by several approaches categorised as physical deposition (e.g., spin coating, dip coating and drop casting), covalent attachment (e.g., layer-by-layer self-assembly) or in-situ polymerisation (e.g., electrochemical deposition).

### 3.1. Optical Sensor Platforms

Optical sensor platforms harness various optical phenomena that occur as a result of the interaction between an analyte and a MIP layer to derive analyte-correlated sensor responses. Generally, optical sensors have high sensitivity; hence, their combination with a target-selective MIP plays a significant role in label-free molecular analysis. The most frequently utilised optical platforms in the field of molecular imprinting are SPR and SERS.

#### 3.1.1. Surface Plasmon Resonance

SPR is a highly sensitive optical system that offers real-time and direct analysis. It monitors and records changes in refractive indices resulting from binding interactions between a target analyte in solution and a receptor immobilised at close proximity to the transducer surface, at which localised electromagnetic waves propagate [83]. The SPR signal that emanates from a shift in the resonance angle corresponding to a change in the surface refractive index can provide information on the amount of bound analyte, affinity of the receptor for the analyte and the association or dissociation constants of the binding interactions [84]. SPR has been widely adapted and utilised in many fields, including molecular engineering, food analysis, clinical diagnosis, proteomics, environmental monitoring, bacteriology, virology, cell biology, and drug discovery. Although the sensitivity of SPR is not in doubt, its versatility was initially restricted to the analysis of large biomolecules, as it suffered a major setback in handling biologically and environmentally important small analytes because the induced change in refractive index is usually too small to generate adequate responses [85]. Due to their robust nature that enables them to withstand harsh chemical conditions of regeneration with no appreciable loss in their activities, MIPs are more adaptive selective recognition layers that can be easily integrated with SPR when compared to antibodies [86]. Such integration has gained wide utilisation in detecting both small and large analytes, accounting for the plethora of publications in this field [87]. Moreover, SPR can be produced in portable miniaturised forms, thus accentuating its utilisation by numerous research groups for different applications [88].

Films and nanoparticles are the most common MIP formats used in fabricating MIP-based SPR sensors. In what is probably the first report of antibiotic determination by a MIP film on a lab-on-chip label-free optical platform, Frasconi et al. [89] studied the detection of three members of aminoglycoside antibiotics, kanamycin, streptomycin and neomycin, using a composite film consisting of bisaniline-cross-linked AuNP and boronic acid ligand. By harnessing the reversible association between vicinal diols’ functionality of the target molecules and boronic acid of the ligand, a selective MIP layer was formed on the electrode by the electropolymerisation of the thioaniline monomer in the presence of the target molecules. Following template removal, the resulting SPR sensor displayed a high sensitivity indicated by a low limit of detection, LOD (0.2 to 2 pM), as well as good selectivity.

In an attempt to fabricate a sensitive, and rapid response MIP-based sensor capable of remote sensing, a localised and propagating SPR based fibre optic sensor for tetracycline was fabricated by a sequential coating of a multimode optical fibre, with layers of silver film and nanoparticle prior to the coating with tetracycline MIP film prepared by thermal polymerisation [90]. Analyte rebinding was evaluated by monitoring the concentration-dependent absorption spectra of a polychromatic light, measured by a spectrometer attached to the other end of the fibre. Following characterisation, the sensor demonstrated a higher sensitivity than a system solely based on localised SPR and was accompanied by an LOD of 2.2 nM and linearity ranging from 10 nM to 10 μM.

In a report by Ayankojo et al. [91], an SPR chip was modified with a MIP film prepared by the sol-gel technique and used for detecting amoxicillin, a beta-lactam antibiotic. Combining organic and inorganic polymers, an optically transparent hybrid MIP film was endowed with an increased porosity; hence, a larger surface area was generated. After optimisation, the sensor demonstrated high sensitivity and selectivity for amoxicillin and was able to detect the analyte down to the pM range (LOD of 73 pM). Moreover, the sensor demonstrated a good recovery in tap water (93–96%) and retained its capacity after 6 months of storage at room temperature.

Moreover, Sari et al. [92] reported the determination of ciprofloxacin in water using an SPR integrated with MIP nanoparticles, synthesised by two phase miniemulsion polymerisation of methacrylic acid. An LOD of 3.21 and 7.1 ppb was obtained in ultrapure water and synthetic wastewater, respectively, with a recovery of about 87%, thus demonstrating its potential use in antibiotic pollutant analysis.

#### 3.1.2. Surface-Enhanced Raman Spectroscopy

SERS is a non-invasive optical label-free detection method that offers sensitive and rapid analyte determination by directly monitoring the Raman spectra assigned to an adsorbed molecule on the SERS surface. SERS enables amplification of typical Raman signals by employing nanometallic surfaces (e.g., Au or Ag) that could be excited by electromagnetic radiation to generate a localised SPR. Molecular adsorption within the vicinity of this electron field causes an enhancement in the Raman signal up to several orders of magnitude, and thus allows analyte detection down to low concentrations [93]. The active metallic surface of SERS when integrated with MIPs provides the opportunity to obtain sensitive and selective signals by indicating the characteristic vibrational spectra of the analyte adsorbed on the MIP-modified surface. An advantage of SERS over SPR is its lack of interference or disturbance arising from non-specific adsorption on random polymer sites and insensitivity to the influence of polymer swelling or shrinking and pH [94]. To integrate MIPs with SERS, chemical functionalisation of the metallic surface is usually performed. Notwithstanding, real-world application of SERS sensors suffers a major setback, arising from the lack of reliable and cost-effective substrates that hampers its consistent performance [95]. Efforts devoted to overcoming this challenge and to advance this field are put together in a recent review [96].

The first SERS-MIP was reported by Kostrewa et al. [94] in which Au or Ag metallic film layers were deposited on a glass slide as the SERS active substrate that was subsequently modified with MIP using N-benzyloxycarbonyl-(L)-aspartic acid, N,N1-diethyl-4-vinylbenzamidine and EDGMA as the template, monomer and cross-linker, respectively, in a porogenic solvent of tetrahydrofuran. Although the performance of such a combination was promising, the MIP polymer material lacks robust stability on the underlying metallic substrate. Following this first report, technological advancements have been made to date in the design and use of MIP-SERS for chemosensing by considering different modes of sensor construction, mechanisms for signal enhancement for various applications, etc. Thus, a multibranched gold-silica-core-shell MIP nanocomposite possessing improved sensing properties was synthesised on a SERS substrate for enrofloxacin recognition. Analyte binding results in the enhancement of the Raman signal by the Au substrate acting as a localised SPR source. Following optimisation, the sensor demonstrated high selectivity, a much lower LOD (1.5 nM) than previous reports and adequate performance in water and food samples [97].

In other research, cloxacillin was analysed using SERS substrates modified with vertical gold-capped silicon nanopillars and integrated with magnetic MIP nanoparticles made via the core-shell technique, in which a cloxacillin MIP layer was deposited on an iron oxide microsphere core using free radical polymerisation. Following Soxhlet extraction of the template, the sensor indicated an LOD of 7.8 pM and good recoveries between 85 and 126% [95]. Recent publications detailing the combination of antibiotic MIPs and optical platforms are summarised in Table 2.

### 3.2. Piezoelectric Sensor Platforms

Piezoelectric sensor platforms employ the effect of transformation between the mechanical and electrical properties of a material to derive analyte-dependent signals. This is observed when a piezoelectric material (e.g., quartz, zinc oxide, etc.) is subjected to a mechanical or electrical perturbation, causing it to generate either electrical charges on its two opposing surfaces or a geometrical deformation. Due to their sensitivity, piezoelectric materials are widely applied in sensing and other numerous applications [109]. QCM and SAW are the two most widely utilised piezoelectric platforms in MIP research.

#### 3.2.1. Quartz Crystal Microbalance

QCM is a piezoelectric transducer, where a piezoelectric material (usually monocrystalline quartz) is confined between two metal electrodes (usually Au or Ag). The quartz crystal oscillates in a pure shear mode if an electric field of the appropriate (resonant) frequency is applied to the electrodes. This resonant frequency is influenced by a combination of the thicknesses of the quartz and metallic electrodes, as well as the mass and viscosity of the studied layer adjacent to the electrodes. Thus, if the properties of the layer are altered, QCM is capable of measuring the same by correlating with the changes in its resonant frequency. Sauerbrey’s equation (Equation (1)) establishes the relationship between the resonant frequency of QCM and mass changes in a studied layer attached to its electrode, assuming the deposited layer is rigid and thin.

Δ*f* = −*f*_0_^2^Δ*m*/(*Nρ*) = −*C*_*f*_Δ*m* (1)

Δ*f* is the resonant frequency change (Hz), *f*_0_ is the fundamental frequency of the crystal (Hz), Δ*m* is the mass change (g/cm^2^), *N* is the frequency constant for quartz (167 kHz·cm), *ρ* is the density of quartz (2.65 g/cm^3^); *C_f_* is the sensitivity factor (for 5 MHz quartz crystal, 56.6 Hz·μg^−1^·cm^2^).

Practical applications of QCM range from monitoring film deposition rate to the analysis of kinetics of molecular interaction in a chemo/biosensing system. QCM can be combined with an electrochemical apparatus, in which case an electrochemical QCM (EQCM) system that facilitates a simultaneous evaluation of mass and electrochemical data in a single experiment is obtained. Moreover, QCM could be integrated with a flow injection analysis to form a simple but powerful technique (QCM-FIA) for the in-situ monitoring of the binding interaction between an analyte and a recognition layer in close vicinity to the quartz crystal [110]. Although QCM measurements are mostly carried out at room temperature because of the excellent properties displayed under such conditions, high temperature measurement applications have also been reported [111,112]. However, because the frequency shift is directly proportional to the mass of any physically adsorbed substance on an unloaded QCM, obtaining qualitative information would, thus, demand the input of a selective element, such as MIP [113]. Early-stage researchers combining MIP with QCM depended on self-built systems that are usually prone to external disturbance (e.g., humidity, temperature or pressure) during experimental runs [114]. Nowadays, there exists commercially available stable systems in a miniaturised format that may be further developed for POCT. Thus, the adaptation of MIP with QCM for detecting numerous analytes is commonly reported and reviews on advancements in this study are available [115,116]. However, analysis of reports on the adaptation of these sensors for aqueous environmental monitoring is scarcely presented; hence it would be the focus of this contribution.

Ayankojo et al. [68] demonstrated the possibility to detect amoxicillin on QCM sensors without the use of signal amplifying materials. Following a computational and spectroscopic assisted study of the interaction between the template and monomer, as well as the optimal electrodeposition parameters, the antibiotic imprinted thin film consisting of poly(meta-phenylenediamine) was deposited directly on the transducer surface. The sensor displayed high sensitivity, an LOD down to 0.2 nM and selectivity towards amoxicillin against other antibiotics in aqueous media. However, further characterisation of the sensor’s affinity vis-a-vis the determined LOD value would be beneficial.

In another report, MIP particles prepared by using 3-aminopropyltriethoxysilane (APTES) and tetraethoxysilane (TEOS) as functional and cross-linking monomers, respectively, were combined with QCM for the analysis of enrofloxacin, a fluoroquinolone antibiotic [117]. The immobilisation of the particles was achieved by forming a homogeneous colloid consisting of a mixture of the MIP particles and Nafion solution that was then transferred onto the QCM surface via a flow injection arrangement. Although the sensor’s selectivity was not reported, the fabricated sensor shows good sensitivity, precision, and recovery in different aqueous matrices.

#### 3.2.2. Surface Acoustic Wave

SAW is another piezoelectric sensor platform where an acoustic, mechanical wave propagates, confined to the surface of a cut piezoelectric crystal. Interaction between the surface and an adjacent layer affects the acoustic wave and leads to a corresponding change in the wave velocity and amplitude. The change in velocity, in response to mass changes, is recorded as the frequency shift (phase shift), while amplitude change responds to the change in viscosity of the layer on the surface. SAW is similar to QCM in the use of piezoelectric material but has a distinct advantage of having higher sensitivity, capable of detecting mass changes at either the nanogram or picogram scale [118]. This is due to the surface propagation of the acoustic wave making it more susceptible to influence by surface perturbations as compared to the bulk propagating wave of QCM. In addition, the higher operating frequencies (100 MHz to a few GHz) of SAW contribute to its higher sensitivities.

Another remarkable advantage of SAW technology is the multiplexing capability (combination of multiple sensor elements in a single chip) that allows considerable reduction in experimental time and expenses. Two different modes of acoustic waves are commonly adopted in SAW devices: Rayleigh and Love waves. The Rayleigh acoustic wave appears to be the most commonly used in SAW sensor devices; however, owing to losses from radiation, they are not usually used in liquid operation [119]. Love waves, on the other hand, are adaptable for utilisation in liquid operation because they consist of shear-mode vibrations, and hence are found in many SAW devices adapted for liquid measurements [120,121]. For the purpose of sensing, SAW devices are usually coated with a thin film of molecular receptors including MIPs and have, thus, demonstrated extreme usefulness in the analysis of any kind of target molecules, such as organic vapours [118], inorganic gases [122] and bioanalytes [75,123].

The last decade witnessed an increased interest in the fabrication of MIP-based SAW sensing devices for the determination of antibiotics. A fluoroquinolone antibiotic, flumequine, was determined in aqueous media by a MIP-modified shear horizontal SAW sensor operating at 104 MHz [73]. The rebinding analysis obtained by monitoring the phase shift variations with changes in analyte concentration indicated an LOD of 1 μM. However, little attention was paid to the importance of a reference sensor, which prevented further comprehensive understanding of the sensor performance.

In addition, sulfamethizole, a representative sulfonamide, was imprinted in poly(meta-phenylenediamine) film, electrodeposited on a multi-channel Love-wave SAW sensor device [75]. The four-channel system allows simultaneous analysis of analyte rebinding on both imprinted and its reference non imprinted film, thus reducing errors due to possible variations between measurements and sensor reproducibility. Following optimisation, the sensor demonstrated a remarkable performance in terms of selectivity and low limit of detection in the sub-nanomolar range. Moreover, a reproducible usage of the sensor for up to three rebinding-regeneration cycles was observed.

Moreover, apart from QCM and SAW, there exist other recent reports, detailing the label-free detection of antibiotics utilising other mass sensitive transducers. These include the report by Okan and Duman [124], where they showcased the label-free analysis of erythromycin using a MIP particle based microcantilever mass sensor in air. Although the sensor demonstrated good performance, similar analysis in a liquid environment may constitute a challenge arising from liquid damping. Table 3 summarises the recent publications showcasing the combination of MIPs and piezoelectric sensor platforms.

### 3.3. Electrochemical Sensors

Electrochemical sensors are another category of label-free sensor transducers that have by far gained the highest popularity among researchers in the field of chemo/biosensor fabrication. This wide recognition is owed to their low cost of manufacture, simple experimental set-up, the possibility for easy miniaturisation, ease in achieving wireless control, hence commercialisation, as well as adaptability for a broad range of applications [128,129,130]. Usually, a typical electrochemical sensing system will consist of three electrodes; a working electrode on which the molecular interaction occurs serves as the transduction element, a counter electrode that connects with the electrolytic solution and allows the flow of electrons to and from the working surface and a reference electrode that helps in maintaining and stabilising the specified potential of the electrochemical set-up [131]. The principle of electrochemical detection relies on monitoring changes in electrochemical parameters (e.g., potential, current, impedance or conductance) induced by the presence of an analyte on the transducer surface relative to the other electrodes. Generally, the presence of an analyte could either bring about a change in the redox reactions when the molecule is oxidised/reduced or affect the charge transfer between the transducer and a redox-active solution at close proximity to its surface. Thus, based on the parameter being measured and the conditions of the controlled parameter, electrochemical sensing systems are routinely categorised into potentiometry, amperometry, voltammetry, and conductometry sensors. In a potentiometric sensor, the potential difference between the working and reference electrodes is monitored under a condition of no current flow. Amperometric sensors measure the flow in current between the working and counter electrodes at an applied fixed potential, while voltammetric sensors monitor the current with varied potentials. In a conductometric experiment, a change in the conductivity of the working electrode resulting from obstruction to the charge transfer between it and a probe solution is measured.

When combined with a MIP, electrochemical sensors show high selectivity, easy and cost-effective preparation, as well as suitability for POCT application. Consequently, different analytes have been determined by MIPs immobilised on electrochemical transducers. In label-free molecular imprinting research, electrochemical sensors are by far the most adopted. The technique used for detecting an analyte by an MIP-based electrochemical sensor is dependent on the nature of the analyte itself. Direct oxidation or reduction of electroactive analytes can be induced on the working electrode to generate the electrochemical signal. However, for non-electroactive targets, an external reversible redox probe medium is usually required for the electrochemical setup. In this regard, a negatively charged redox probe, e.g., [Fe(CN)_6_]^3−^/[Fe(CN)_6_]^4−^ is common, but positively charged ones, such as [Ru(NH_3_)_6_]^3+^/[Ru(NH_3_)_6_]^2+^, are also available, depending on the charge expected to accumulate on the MIP-based sensor [132].

Magnetic MIP nanoparticles prepared by co-precipitation around Fe_3_O_4_ particles were immobilised on a carbon SPE for the detection of sulfamethoxazole in aqueous media [133]. The magnetic properties of the sensor help to concentrate the analyte to the MIP surface by selective capture. Electrochemical impedance spectroscopy monitoring of target rebinding indicates an LOD (1 pM) close to that obtained from liquid chromatography and mass spectrometry analysis, as well as good target selectivity. Moreover, the successful utilisation of the sensor was demonstrated in seawater samples. Similarly, the selective determination of sulfamethoxazole in surface water was achieved using a boron doped diamond electrode modified with an electrodeposited MIP film, consisting of a matrix of polypyrrole [134]. Good sensor performance was observed, in which an LOD of 24.1 nM and linearity of 0.1–100 μM were obtained. Moreover, the sensor demonstrated remarkable selectivity for the target against structurally similar antibiotics sulfadimethoxine, sulfadiazine and sulfafurazole and good recoveries (96.0–106.2%).

In another report, ceftizoxime imprinted film integrated with a voltammetry sensor was reported by Beytur et al. [135]. The sensor was prepared by modifying a GCE with a composite of Ag@Au NPs and ionic liquid prior to MIP immobilisation. Although there is a need to further evaluate the analyte rebinding on the sensor, an LOD of 2.0 pM and linearity in the range of 1 nM to 10 pM were obtained in water. The sensor also demonstrated high selectivity, suitable for antibiotic analysis.

**Figure 4:**
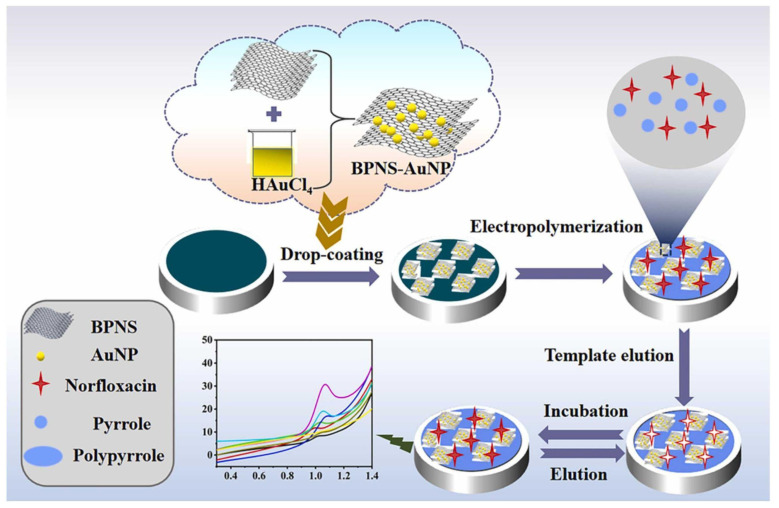
Fabrication of MIP-black phosphorus nanosheet nanocomposite on GCE (MIP/BPNS-AuNP/GCE) for sensitive determination of norfloxacin (NOR). Reprinted with permission from [136]. Copyright 2022 American Chemical Society.

Recently, an ultrasensitive, label-free voltammetric sensor for the selective determination of norfloxacin, based on an Au nanoparticle-functionalised black phosphorus nanosheet nanocomposite (BPNS-AuNP) covered by a polypyrrole-based MIP film, was fabricated, as shown in Figure 4 [136]. The authors found that BPNS-AuNPs exhibited a synergistic catalytic effect towards norfloxacin oxidation, along with providing a large surface area. Moreover, the MIP/BPNS-AuNP/GCE sensor achieved the detection of norfloxacin at the nanomolar level, with an extremely low detection limit, high sensitivity, good selectivity, repeatability, and stability. The sensor was successfully applied to detect norfloxacin in pharmaceutical, food, and environmental samples. A summary of the recent publications in the field of MIP-based electrochemical sensors is shown in Table 4.

## 4. Signal Amplification Approaches for Antibiotic MIP-Based Sensors

Sensitivity is a critical parameter in sensor fabrication. However, achieving a high sensitivity for low molecular weight targets, such as antibiotics, may be challenging because these molecules induce comparatively lower responses to the sensor transducers. Nevertheless, several approaches exist for enhancing the transduction of the chemical interactions between small molecules and a MIP recognition layer immobilised on the sensor. Such approaches aim to either increase the effective surface area by improving porosity, using nanomaterials such as AuNP, AgNP, SWCNT, MWCNT, etc., or increasing the amount of recognition sites created within the polymer. Other methods include improving the signal-to-noise ratio of the sensing system, the incorporation of an ionic liquid and/or a cationic surfactant, e.g., cetyltrimethylammonium bromide [156], etc.

For piezoelectric and other mass-sensitive sensors, signal amplification is usually achieved by increasing the transducer frequency. Consequently, several antibiotic MIPs have been developed on piezoelectric sensors with different operating frequencies, ranging from 5 to 100 MHz [68,73,117,157,158]. Moreover, since small molecules only induce a frequency shift if an adequate amount is integrated onto the transducer, increasing the amount of recognition sites in the polymer is another overriding approach. This is usually achieved by bulk imprinting, where analyte recognition is permitted over the entirety of the bulk of the polymer compared to the surface-restricted recognition, but this leads to a longer response time and needs to find a balance between sensitivity and speed of the resulting sensor [113,159]. Herein, the molecular template does not only shape the sterical and chemical qualities of the binding sites but also establishes the diffusion path for easy access to the binding sites. In addition, MIP nanoparticles could be prepared by precipitation polymerisation and used as a recognition layer on QCM for the sensitive determination of antibiotics, e.g., penicillin and ampicillin [160]. The MIP format in such a sensor would increase the effective surface area, thereby improving the sensitivity of detecting the analytes at relevant concentrations.

For the analysis of small molecules on SPR, an inhibition assay was often used, where small analytes are premixed with antibodies and unbound antibody sites are captured by the small analytes immobilised on a sensor surface [161]. Other prevailing alternative approaches involve signal enhancement via the use of nanoparticles, in particular gold nanoparticles [162], increasing surface loading of receptor elements by coating the sensor surface with a matrix layer, e.g., dextran, while providing a favourable environment for molecular interactions [159].

By coupling a localised plasmon generated on AuNPs with the surface plasmon of the transduction system (LSPR), the signal induced by binding events on the SPR chip can be amplified. Such a possibility was demonstrated by Frasconi et al. [89], where AuNP was functionalised with a capping layer of thioaniline monomer and boronic acid ligand to amplify the low refractive index changes that occur upon rebinding of the small molecular weight analytes (e.g., kanamycin, streptomycin and neomycin) on MIP modified SPR. LSPR provided by the AuNP, coupled with surface plasmon on the transducer, resulted in the high sensitivity of the sensor and detection limit down to the pM range.

**Figure 5:**
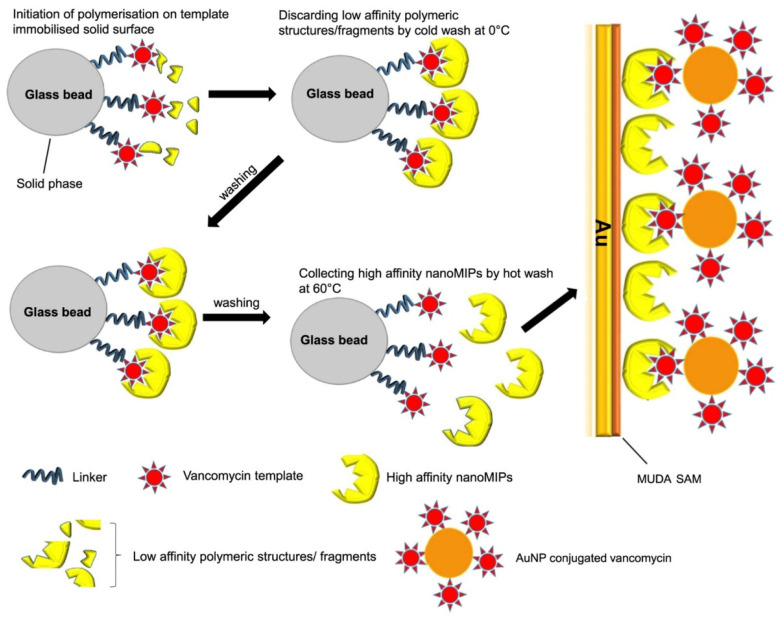
Schematics of the preparation of vancomycin nanoMIPs and their integration on SPR sensors for amplified vancomycin detection, following Au surface functionalisation with 11-mercaptoundecanoic acid self-assembled monolayer. Reprinted with permission from [44]. Copyright 2018 Elsevier.

In addition, a high-affinity nanoMIP was coupled with SPR and used for the selective determination of vancomycin [44]. The MIP nanostructure was prepared by employing solid-phase synthesis, in which UV light-assisted synthesis of the polymer (polyitaconic acid) was achieved around the template immobilised on a glass bead solid phase. This was followed by cold washing to remove low-affinity materials and hot washing to collect high-affinity nanoMIPs (Figure 5). After particle characterisation, a uniformly distributed nanoMIP (average size of 174  ±  2 nm) was covalently immobilised on SPR Au, functionalised with an amine terminated 11-mercaptoundecanoic acid and was successfully utilised in the selective target analysis. Furthermore, due to the success being recorded in the development of optical fibre sensors, they are gradually becoming a substitute for the traditional SPR substrates [163]. This is primarily due to the intrinsic total internal reflection properties of optical fibres that prevent significant loss in signals, hence improved sensitivity.

**Figure 6:**
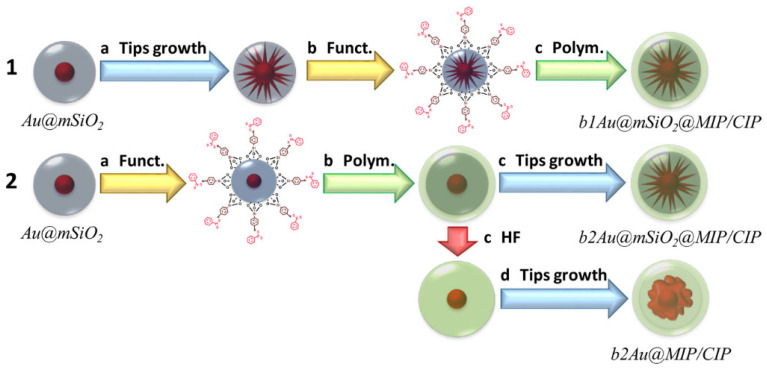
Schematic representation of the different mesoporous nanocomposites fabricated for the development of SERS nanosensors. Reprinted with permission from [97]. Copyright 2022 American Chemical Society.

The signal amplification on SERS is mostly achieved by the incorporation of nanomaterials. For example, in an attempt to override the slower binding kinetics and difficulty in transforming antibiotic binding events into a measurable signal, Carrasco et al. prepared a mesoporous MIP composite (Figure 6) consisting of multibranched gold-silica-MIP core-shell nanoparticles as a label-free nanosensor for the detection of enrofloxacin by SERS [97]. The nanosensor showed improved sensing properties through the amplification of the Raman scattering induced by the binding of the antibiotic molecules, thus lowering LOD to 1.5 nM. Similarly, Ashley et al. [95] integrated a magnetic MIP on SERS substrates modified with vertical gold capped silicon nanopillars for cloxacillin detection. The use of the magnetic MIP nanoparticles provided magnetically susceptible and good target-selective properties to the sensor. The sensor shows a good selectivity, an LOD of 7.8 pM and recovery ranging from 85 to 126%.

Because of its vast application in environmental monitoring, electrochemical sensors have been used in a variety of signal amplification schemes for antibiotic determination. A streptomycin imprinted poly(pyrrole-3-carboxylic acid) film was prepared on GCE that was previously modified with electrochemically reduced graphene oxide for the determination of streptomycin. Although a kinetic study was not indicated, the sensor demonstrated improved performance with an LOD of 0.5 nM and good recoveries between 95.8 and 107% [164].

CNTs combined with Cu nanoparticles were used to modify a GCE as a functional affinity structure, when integrated with a MIP film prepared from MAA and EGDMA by the sol-gel technology used in chloramphenicol detection. Herein, the sensor response induced by the analyte binding on the MIP would be amplified by the CNT-metal NP composite. The sensor demonstrated improved sensitivity and selectivity compared to other methods and up to 10 times reusability [45]. In other research, a polypyrrole-based MIP was prepared on GCE to generate a conductometry sensor for the sensitive and selective analysis of chloramphenicol [165]. To improve the surface area and charge transfer, thereby increasing the sensor sensitivity and performance, the WE of GCE was modified with zirconium-based metal organic framework and carbon dots. Following the careful optimisation, an LOD of 0.061 pmol/L and linearity in the range of 0.1–100 pmol/L were achieved. Furthermore, the composite-modified electrode demonstrated better performance in terms of improved MIP loading capacity, charge transfer and sensitivity.

**Figure 7:**
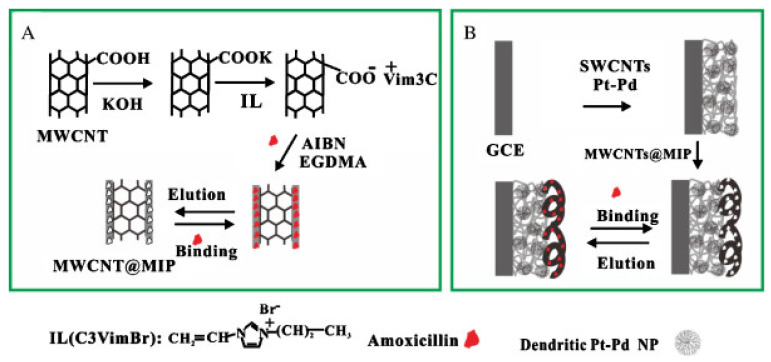
Fabrication protocol for amoxicillin MIP-based sensor using a combination of (**A**) MWCNT, (**B**) SWCNT, nanoparticles and ionic liquid, for sensitivity enhancement. Reprinted with permission from [167]. Copyright 2015 Elsevier.

Furthermore, to enhance the sensitivity of an MIP-based screen-printed carbon electrode towards tetracycline, Devkota et al. incorporated AuNP and sodium dodecyl sulphate (SDS), an anionic surfactant, during the electrochemical synthesis of molecularly imprinted overoxidised polypyrrole on the working electrode [166]. Following the template elution by the application of an electrochemical potential, an increase in the electrode sensitivity was observed. SDS participates in the sensitivity enhancement by interacting with the target cationic antibiotics, with its polar ends oriented towards the solution in a monolayer assembly. However, in another report, Yang and Zhao [167] demonstrated the sensitive detection of amoxicillin, employing a combination of MWCNTs, SWCNT, dendritic Pt-Pd nanoparticles and ionic liquid to enhance the signal induced on the MIP-coated GCE (see Figure 7). The presented approach yielded a wide linear range between 1 nM and 6 μM and LOD of 89 nM.

**Figure 8:**
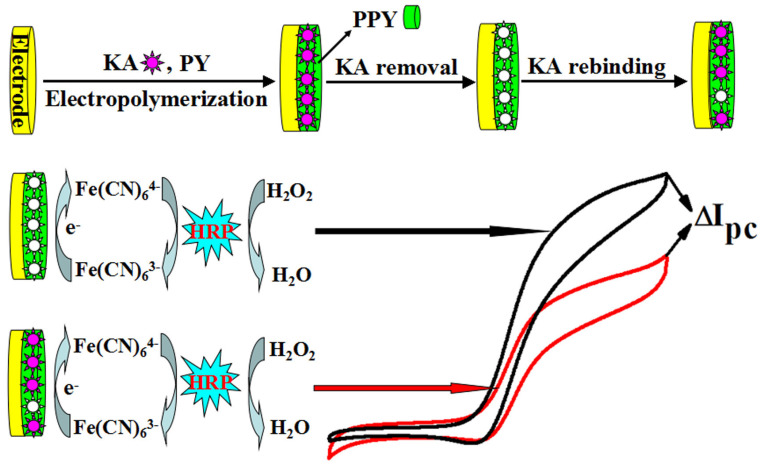
Fabrication protocol for amoxicillin MIP-based sensor using a combination of MWCNT, SWCNT, nanoparticles and ionic liquid, for sensitivity enhancement. Reprinted with permission from [168]. Copyright 2015 Elsevier.

Another approach for improving the sensitivity of electrochemical MIP-based sensors, especially for electro-inactive targets, was reported by Lian et al. MIP sensing was coupled with bioelectrocatalysis at the MIP-modified electrode surface for signal amplification [168]. This method involves a horseradish peroxidase catalysed electrochemical reduction of peroxide, mediated by a K_3_[Fe(CN)_6_] redox marker on the electrode (Figure 8). When used for detecting kanamycin antibiotics, this intervention brought about an eight-fold increase in the sensor response and two orders of magnitude lower LOD value. The approach was found not only useful for other antibiotics but also suitable with other bioelectrocatalysis systems.

Other increasingly adopted signal amplification approaches for antibiotic imprinting that were not critically discussed in this review include the adaptation of metal organic framework, aptamer, or photonic crystals and their integration with MIPs, as well as electrode modification with composites of novel nanomaterials, e.g., gold nano-urchins and graphene oxide that present emerging unique properties [79,169,170,171,172].

## 5. Practical Challenges of Antibiotic MIP

Without a doubt, MIPs represent a promising alternative to biological elements in sensor development. Although MIPs still suffer certain setbacks in comparison to antibodies, in relation to the binding affinity and kinetics, thus depicting a potential limitation to the analysis of very low analyte concentrations under certain conditions, their potential adoption in several applications e.g., sensing, cannot be overemphasised. However, despite the great potential, the commercialisation of MIP-based sensors is still far from being realised. Although there exist some commercial MIPs that are available from Sigma Aldrich (now Merk) or AFFINISEP, they are only suitable for applications involving separation (including antibiotics separation) by solid-phase extraction, whereas MIP-based sensors ideal for onsite monitoring of environmental contaminants, e.g., antibiotics or medical diagnostics, are difficult to find. Typically, commercialising a lab-based technology is not a straightforward process, but this constitutes a significant hurdle to be crossed if MIP-based sensors are to be translated to the market. To facilitate progress towards the achievement of this aim, there is an increasing number of patents and patent applications on MIP-based sensors for antibiotics analysis. A quick search on the Espacenet database returns 13 such patents within the last 5 years. However, despite concerted efforts and the accompanying success witnessed in surpassing fabrication technicalities arising from the need for portability, usability and cost of MIP-based sensors, the challenges encountered in its design and applications must be overcome to adequately compete with commercially available sensors and diagnostic devices.

One of the main drawbacks to the commercial implementation of antibiotic MIP, as well as other MIPs, is the incomplete elution of the template during MIP preparation, thereby causing template leakage during MIP usage. Generally, it is perhaps difficult to ensure a 100% template extraction from the polymer. Thus, during MIP-based sensor utilisation, the entrapped template is being released continuously from the MIP and interacts with target sensing, affecting assay accuracy, especially for the trace analysis of small molecules, such as antibiotics. In addition, low selectivity may arise due to an incomplete template removal that further prevents efficient analyte detection in complex environmental samples. This effect could be more pronounced for MIPs designed by covalent imprinting, where the covalent bond may lead to a slower template removal. Therefore, possible maximum template removal is an obligatory step in MIP preparation. However, it was found that not many reports focus on optimising this parameter during MIP preparation. To overcome this challenge, the use of dummy templates bearing close resemblance in size and shape to the targets is a common overriding strategy [173]. In addition, the electrochemically-assisted removal of the template molecules [166] has been reported to achieve improved elution by eliminating cationic charges from the matrix, leading to the escape of the template anion. Although, this may further raise questions relating to its broad applicability, as well as binding site geometry. Above all, the use of a sufficient amount of crosslinkers that preserve adequate porosity and remove difficulties in template removal during the washing out process is essential.

On the other hand, the performance of MIP prepared on the basis of noncovalent imprinting may also be affected when used in aqueous samples, where water affects H-bonds between the template and functional monomer, thereby disrupting the binding equilibrium and limiting the practical application of the MIP-based antibiotic sensor. To circumvent such limitations, the use of organic solvents during MIP synthesis prior to rebinding in an aqueous environment is conventional. However, such an approach may also result in some compromise in the performance of the MIP-based sensor, since it was found that the use of the same solvent during synthesis and rebinding is critical for optimal performance [174]. Other suggestions include giving attention to monomers hydrophophilicity during functional monomer selection, adopting the semi-covalent imprinting that allows covalent interactions between functional monomer and template prior to polymerisation, whereas analyte rebinding is governed by noncovalent interactions, and controlling polymer surface modification by adopting a controlled radical polymerisation [48].

Moreover, for a reliable characterisation of the performance of an MIP-based chemosensor, a homogeneous distribution of the binding sites within the polymer that is uniformly immobilised on the transducer is required. However, MIPs have a certain degree of heterogeneity in the binding sites, arising from the preparation protocol. The polymer matrix, by nature, can adsorb the target molecules through weak interactions. This allows for additional contribution from non-specific binding to the obtained signal, hence the variation in the binding constants [175]. While the challenge with non-specific binding still remains, large-scale reproducibility of MIP layers on electrode transducers is ensured by focusing on electropolymerisation, dip or roll-coating, surface grafting, etc., to prevent variation from batch to batch [39].

Lastly, another conceivable obstacle could be the challenge of using these materials in the media where the target antibiotics are naturally found, without the need for a laborious sample preparation step. This is essential in the consideration of the possible incompatibility and/or cross-reactivity of the matrix components. Therefore, vigorous and continual efforts are still needed in MIP research to achieve the aim of commercialised MIP-based sensors in the shortest space of time.

## 6. Conclusions and Future Perspectives

The increasing threats of antimicrobial resistance has accentuated the call for robust sensing systems that combine both sensitive and selective properties, as well as portability. Certainly, MIP presents a convincing alternative to natural biomolecules as receptor layers for sensors. Their capacity to facilitate the fabrication of recognition elements for analytes with no natural receptors makes them appealing for designing monitoring devices for multidisciplinary applications. This explains the increase in research attention and scientific reports in the development of MIP-based sensors as environmental monitoring tools for antibiotic pollutants. However, despite the copious potential benefits of fabricating antibiotic MIP-based sensors, their commercial implementation has suffered significant setbacks mostly related to template leakage, due to incomplete template removal, complications in fabricating robust and homogeneous MIPs on electrodes, as well as the challenges of mass production and real sample analysis without laborious sample pretreatments. This review evaluates the significant efforts towards the development of commercially viable antibiotic MIP sensors.

To ensure preparation simplicity, performance accuracy and ease of usage, several studies have focused on sensing platforms, including optical (e.g., SPR and SERS), piezoelectric (QCM and SAW) and electrochemical transducers that support label-free molecular detection and that can be miniaturised. Among these, electrochemical sensors were revealed as the dominant platform on which antibiotic MIPs are frequently integrated, suggesting their low production cost, ease of operation, portability, as well as their potential for realising commercialised POCT devices. The sensors examined here largely demonstrated sensitive and selective capacities to detect antibiotic pollutants in real samples at the relevant concentration levels, thanks to the valuable computational approach that assists in efficient functional monomer selection and the various signal amplification methods that enhance sensitivity.

Following technological advancement, publications describing computational studies of molecular imprinting systems for pre-polymerisation have increased significantly. The computational tool has enhanced experimental optimisation, allowing appropriate selection of the type and molar amount of functional monomer and crosslinker for a specific template. However, not much detailed attention has been given to possible template–template interactions and their significance to a successful imprinting process and subsequent target recognition [40]. This is particularly critical where noncovalent interaction is chiefly employed for template imprinting. In addition, only a few studies probe the fate of functional monomer–template complexes during polymerisation. Such information is essential to the advancement of the knowledge of the mechanism governing MIP formation and consequent efficiency of an MIP-based sensor.

The performance of MIP-based sensors described in this review was compared with those of conventional chromatography techniques for antibiotic analysis in water samples. The result demonstrated that although the reported LOD values of MIP-sensors range from 103 to 105 nM, the lowest values are still comparable with the LOD values reported for the HPLC technique (0.031–0.047 nM) [176]. Moreover, an even lower LOD value of 107 nM was reported for the molecularly imprinted two-dimensional photonic crystal hydrogel sensor for the label-free recognition of sulfamethoxazole [105]. Nevertheless, despite these promising results achieved for MIP sensors on the laboratory scale, the large-scale use is still problematic, due to several challenges in the synthesis (see Section 5), mass-scale production and commercialisation.

Moreover, to overcome some of the challenges of antibiotic MIP-based sensor commercialisation, certain compelling solutions were identified. Firstly, the implementation of nanoMIPs being developed by MIP Diagnostics Ltd. represents a viable solution, provided a robust integration with the sensor transducers is achievable. In addition, the adoption of disposable multiplexing transducers, such as SPEs, in combination with mobile controllable miniaturised electrochemical devices, would further accelerate efforts toward the actualisation of portable antibiotic monitoring devices.

Thus, the fabrication of MIP-based label free sensors for antibiotic monitoring channels a novel path in the development of portable devices for environmental screening. Such sensors have demonstrated compelling performances in terms of sensitivity and selectivity as revealed by this review; hence, the adaptation of the outstanding strategies identified herein could help in achieving scalable antibiotic MIP-based monitoring devices.

## Figures and Tables

**Table 2:** Antibiotic MIPs combined with optical label-free sensor platforms and their performance.

Sensor Platform	Target Antibiotic	MIP Format	Media	LOD (nM)	Ref.
SPR	Kanamycin, streptomycin and neomycin	Film	-	0.2 × 10^−3^−2 × 10^−3^	[89]
	Tetracycline	Film	-	2.2	[90]
	Ciprofloxacin	Nanoparticle	Synthetic wastewater	3.21 and 7.1 ppb	[92]
	Erythromycin	Nanoparticle	Water	400	[98]
	Amoxicillin	Film	Tap water	0.073	[91]
	Spiramycin	Membrane	Water	0.027	[99]
	Ciprofloxacin, moxifloxacin, ofloxacin	Nanoparticle	River water	55, 1, 12.5	[100]
	Oxytetracycline	Magnetic halloysite nanotube	Aquaculture wastewater, river water	8.1	[101]
	Ciprofloxacin	Nanoparticle	Fish pond and river water	6860	[102]
SERS					[94]
	Enrofloxacin	Nanoparticle	-	1.5	[97]
	Cloxacillin	Nanoparticle	-	0.0078	[95]
	Enrofloxacin hydrochloride	Nanoparticle	Water	0.0078	[103]
Optical nanocrystalline cellulose	Sulfanilamide	Film	Water	-	[104]
Photonic crystal	Sulfamethoxazole	Film	Tap water	1 × 10^−7^	[105]
	Levofloxacin	Film	Tap water	1 × 10^−3^	[106]
UV—Vis	Enrofloxacin hydrochloride	Nanoparticle	Water	0.012	[107]
	Tetracycline	Nanoparticle	Water	28	[108]

**Table 3:** Antibiotic MIPs combined with piezoelectric label-free sensor platforms and their performance.

Sensor Platform	Target Antibiotic	MIP Format	Media	LOD (nM)	Ref.
QCM	Amoxicillin	Film	Tap water	0.2	[68]
	Enrofloxacin	Nanoparticle	Food	147.5	[117]
	Penicillin and amoxicillin	Film	Water	0.25 × 10^6^−0.30 × 10^6^	[125]
	Chloramphenicol	Film	-	0.74 × 10^3^	[126]
	Chloramphenicol	Nanoparticle	-	177 × 10^3^	[127]
SAW	Flumequine	Film	-	1000	[73]
	Sulfamethizole	Film	Tap water	0.9	[75]
Microcanti- lever mass sensor	Erythromycin	Nanoparticle		1000	[124]

**Table 4:** Antibiotic MIPs combined with electrochemical label-free sensor platforms and their performance.

Sensor Platform	Target Antibiotic	MIP Format	Media	LOD (nM)	Ref.
Au electrode	Lomefloxacin	Film	River and lake water	0.2	[137]
	Erythromycin	Film	Tap water	0.1	[138]
	Chloramphenicol	Film	Aquaculture water	1.24	[139]
GCE	Ceftizoxime	Film	Water	2.0 × 10^−3^	[135]
	Kanamycin	Nanoparticle	Tap water, ground water	1.87	[140]
	Sulfadimethoxine	Film	Aquaculture water	40	[141]
	Sulfanilamide	Film	Lake water	2.30 × 10^3^	[142]
	Ciprofloxacin	Nanoparticle	Tap water	0.21 × 10^3^	[143]
	Enrofloxacin	Nanoparticle	Lake water	0.027	[144]
	Enrofloxacin	Nanoparticle	Water	0.9 × 10^−3^	[145]
Polyvinyl chloride (PVC) membrane	Moxifloxacin	Nanoparticle	Aqueous media	-	[146]
Boron doped diamond electrode	Sulfamethoxazole	Film	Lake water	24.1	[134]
Carbon electrode	Sulfonamide	Nanoparticle	Sea water	10^−3^	[133]
	Azithromycin	Film	Sewage/ wastewater	0.12 × 10^3^	[147]
	Amoxicillin	Nanoparticle	River water	0.75 × 10^3^	[148]
	Furazolidone	Film	River and tap water	0.03 × 10^3^	[149]
	Azithromycin	Film	River and tap water	0.08 × 10^3^	[76]
	Amoxicillin	Film	-	0.54	[150]
Handmade with carbon ink	Sulfadiazine	Film	Water	4.22 × 10^3^	[151]
Indium tin oxide electrode	Chloramphenicol	Film	Tap water	1.8	[152]
	Norfloxacin	Film	Tap water	0.04	[153]
Fluorine-doped tin oxide	Oxytetracycline	Nanoparticle	River and tap water	0.1	[154]
	Chloramphenicol	Film	Tap water	9.35 × 10^−3^	[155]
Black phosphorus nanosheet nanocomposite	Norfloxacin	Film	Water	0.012	[136]

## Data Availability

Not applicable.
